# Human Fetal Brain Connectome: Structural Network Development from Middle Fetal Stage to Birth

**DOI:** 10.3389/fnins.2017.00561

**Published:** 2017-10-13

**Authors:** Limei Song, Virendra Mishra, Minhui Ouyang, Qinmu Peng, Michelle Slinger, Shuwei Liu, Hao Huang

**Affiliations:** ^1^Shandong Provincial Key Laboratory of Mental Disorders, Research Center for Sectional and Imaging Anatomy, Shandong University School of Medicine, Jinan, China; ^2^Radiology Research, Children's Hospital of Philadelphia, Philadelphia, PA, United States; ^3^Cleveland Clinic Lou Ruvo Center for Brain Health, Las Vegas, NV, United States; ^4^Department of Radiology, Perelman School of Medicine, University of Pennsylvania, Philadelphia, PA, United States

**Keywords:** fetal brain connectome, structural network, brain development, middle fetal stage, migration pathway, white matter fibers, diffusion tensor imaging

## Abstract

Complicated molecular and cellular processes take place in a spatiotemporally heterogeneous and precisely regulated pattern in the human fetal brain, yielding not only dramatic morphological and microstructural changes, but also macroscale connectomic transitions. As the underlying substrate of the fetal brain structural network, both dynamic neuronal migration pathways and rapid developing fetal white matter (WM) fibers could fundamentally reshape early fetal brain connectome. Quantifying structural connectome development can not only shed light on the brain reconfiguration in this critical yet rarely studied developmental period, but also reveal alterations of the connectome under neuropathological conditions. However, transition of the structural connectome from the mid-fetal stage to birth is not yet known. The contribution of different types of neural fibers to the structural network in the mid-fetal brain is not known, either. In this study, diffusion tensor magnetic resonance imaging (DT-MRI or DTI) of 10 fetal brain specimens at the age of 20 postmenstrual weeks (PMW), 12 *in vivo* brains at 35 PMW, and 12 *in vivo* brains at term (40 PMW) were acquired. The structural connectome of each brain was established with evenly parcellated cortical regions as network nodes and traced fiber pathways based on DTI tractography as network edges. Two groups of fibers were categorized based on the fiber terminal locations in the cerebral wall in the 20 PMW fetal brains. We found that fetal brain networks become stronger and more efficient during 20–40 PMW. Furthermore, network strength and global efficiency increase more rapidly during 20–35 PMW than during 35–40 PMW. Visualization of the whole brain fiber distribution by the lengths suggested that the network reconfiguration in this developmental period could be associated with a significant increase of major long association WM fibers. In addition, non-WM neural fibers could be a major contributor to the structural network configuration at 20 PMW and small-world network organization could exist as early as 20 PMW. These findings offer a preliminary record of the fetal brain structural connectome maturation from the middle fetal stage to birth and reveal the critical role of non-WM neural fibers in structural network configuration in the middle fetal stage.

## Introduction

From the middle fetal stage until birth, complicated molecular and cellular processes take place in a spatiotemporally heterogeneous and precisely regulated pattern (e.g., Johnson et al., 2009; Miller et al., 2014) in the human fetal brain, yielding not only dramatic morphological and microstructural changes, but also macroscale connectomic transitions. One of the most characteristic fetal brain developmental processes is the migration of neurons from the ventricular zone to the cortical plate along the glial fibers (Rakic, 1972, 1995; Sidman and Rakic, 1973). Most neurons are generated at the ventricular zone, migrate along the glial fibers into the cortical plate, and begin to grow their axonal, dendritic, and synaptic projections in the cortical plate (e.g., Rakic, 1972, 1995; Sidman and Rakic, 1973; Kostović and Rakic, 1990; Innocenti and Price, 2005; Lui et al., 2011). The migration pathways formed by glial fibers usually take place across the cerebral wall, which is a prominent compartment of the human fetal brain and can be typically divided into multiple layers from near the ventricle to the outermost cortical plate (e.g., Kostović et al., 2002; Huang et al., 2013; Yu et al., 2016). In parallel to those maturational processes, human fetal brain white matter (WM) axons appear (Bayer and Altman, 2004, 2005), and structural connections based on axons emerge during this period. As the underlying substrate of fetal brain structural network, both dynamic neuronal migration pathways and rapid developing fetal WM fibers could fundamentally reshape the fetal brain connectome. The brain connectome is believed to play a vital role for structural integration and functional specification of brain systems (Passingham et al., 2002; Sporns et al., 2005). The proper “wiring” of the developmental human brain is therefore critical in developing normal mental functions. Disruptions of the normal maturational processes could be associated with neurodevelopmental disorders such as autism (e.g., Hazlett et al., 2017). Quantifying structural connectome development can, therefore, not only shed light on brain reconfiguration in this critical yet rarely studied developmental period, but also reveal alterations of the connectome under neuropathological conditions. However, such wiring dynamics have not been quantitatively delineated from the middle fetal stage to birth.

Diffusion MRI (dMRI) measures the signal changes caused by water molecule diffusion. Diffusion tensor MRI (DT-MRI or DTI) (Basser et al., 1994), adopting a tensor model and based on dMRI, has been widely used for characterizing the microstructure of developing human brain. Specifically, DTI-based metrics, such as fractional anisotropy (FA) (Pierpaoli and Basser, 1996; Beaulieu, 2002), have been used to effectively quantify the microstructure of the WM fibers of developing human brains. DMRI-based tractography, such as the line propagation tracking method (Mori et al., 1999), has been used to trace neural fibers non-invasively. Based on dMRI tractography, major WM tracts and other brain fibers (such as those in ganglionic eminence in human fetal brain) have been delineated in human fetal (e.g., Huang et al., 2006, 2009; Kasprian et al., 2008; Huang, 2010; Takahashi et al., 2012; Huang and Vasung, 2014; Mitter et al., 2015a,b; Ouyang et al., 2015) and neonatal brains (e.g., Mishra et al., 2013; Ball et al., 2014; Pandit et al., 2014).

The human brain's structural wiring can be quantified with macroscale graph analysis. To quantify a structural network, a connectivity matrix needs to be established. In the connectivity matrix, the network nodes are parcellated brain regions and the network edges connecting the nodes are the traced neural fibers. Graph theory analysis (Rubinov and Sporns, 2010) will then be applied to the connectivity matrices to reveal the quantified network properties. Developmental brain structural networks have been quantified using dMRI-based tractography and graph theory analysis in varying developmental periods from birth to young adults (e.g., Hagmann et al., 2010; Yap et al., 2011; Huang et al., 2015). Information regarding fetal brain networks is difficult to obtain. So far the fetal brain network properties have been delineated with preterm brain MRI as early as the beginning of the 3rd trimester (e.g., Fransson et al., 2007; Smyser et al., 2010; Tymofiyeva et al., 2012; Ball et al., 2014; Brown et al., 2014; van den Heuvel et al., 2015; Cao et al., 2017) and with *in-utero* fetal brain MRI as early as the 2nd trimester (Schöpf et al., 2012; Jakab et al., 2014; Thomason et al., 2014, 2015; van den Heuvel and Thomason, 2016). As revealed by histology (Bayer and Altman, 2004, 2005) and DTI (Huang et al., 2006, 2009; Huang, 2010; Takahashi et al., 2012; Huang and Vasung, 2014), dramatic anatomical changes that could significantly reshape the fetal brain connectome take place in fetal development. However, the human fetal brain connectome from the middle fetal stage to birth has rarely been investigated. The emergence of fetal brain structural connectome remains largely unknown.

We aimed to understand the fetal brain structural network dynamics and the underlying neural fiber contributions to the network configuration. With few long-range association fibers, such as inferior or superior fronto-occipital fasciculus, emerging in the early to the middle fetal stages (Kostović and Jovanov-Milošević, 2006; Huang et al., 2009; Vasung et al., 2010; Takahashi et al., 2012), brain fibers other than fetal WM tracts could play a vital role in early configuration of fetal brain network at 20 postmenstrual week (PMW) (Engle and American academy of pediatrics committee on fetus and newborn, 2004). There are several challenges on quantifying fetal structural networks through integrating dMRI tractography and graph theory analysis from the middle fetal stage to birth. First, the fetal brain surface around 20 PMW is quite smooth and there is no widely recognized method to parcellate smooth fetal brains. Secondly, different from the postnatal human brain (including neonatal brain born at term), the structural connectivity of the human fetal brain is not exclusively contributed to by WM fibers. A relatively large portion of organized neural fibers in fetal brains are neuronal migration pathways. Thirdly, it is difficult to obtain an evaluable DTI dataset of the brains at the middle fetal stage. Here, we acquired DTI data from 10 postmortem fetal brain specimens at 20 PMW, 12 *in vivo* brains at 35 PMW, and 12 *in vivo* brains at 40 PMW. DTI-based tractography and brain parcellation were conducted for all datasets to understand how the fetal brain structural network matures from the middle fetal stage to birth. Graph theory analysis was conducted with brain nodes generated from a template-free algorithm (Leopardi, 2006) and edges quantified based on DTI tractography. The network metrics were computed for all brains to quantify the brain network topological changes during these cross-sectional ages and evaluate whether the small-world property exists as early as the middle fetal stage. Additionally, the traced neural fibers of 20 PMW fetal brains were categorized into two groups based on the fiber terminal locations with group1 fibers having both terminals located in the cortical plate and group2 fibers having one terminal located in the inner layer of the cerebral wall. We measured the contributions of group1 fibers only, group2 fibers only and combined group1 and group2 fibers to the 20 PMW fetal brain network configuration.

## Materials and methods

### *In vivo* neonate subjects and *ex vivo* human fetal brain specimens

Data from 24 *in vivo* neonate subjects and 11 *ex vivo* specimens (see Yu et al., 2016) were acquired. One *ex vivo* specimen was excluded from this study since the traced fiber number from the whole-brain DTI tractography of this specimen was significantly less than those traced from other 10 *ex vivo* specimens, suggesting possible fixation or tissue damage. All 34 datasets from 24 *in vivo* neonate subjects and 10 *ex vivo* specimens were divided into three groups based on postmenstrual age, with the first group around the middle fetal stage (age range at scan 19.1–20.9 PMW, 20.1 ± 0.76 PMW) including 10 *ex vivo* fetal brain specimens, the second group around the middle of 3rd trimester (age range at scan 34.3–35.9 PMW, 35.1 ± 0.45 PMW) including 12 *in vivo* preterm neonate subjects, and the third group around term (age range at scan 40.3–41.6 PMW, 40.7 ± 0.38 PMW) including 12 *in vivo* term-born neonates. All *ex vivo* human fetal brain tissues were borrowed from the University of Maryland Brain and Tissue Bank (BTB) for Developmental Disorder (NICHD contract no.N01-HD-9-0011). These specimens had no detectable morphological abnormalities, as ensured by tissue bank record. *In vivo* preterm and term neonates were recruited from Parkland Hospital in Dallas. These subjects were a part of the cohort for investigating normal prenatal and perinatal brain development and were selected after rigorous screening procedures conducted by a neonatologist and an experienced pediatric radiologist, based on the subjects' ultrasounds, clinical MRIs, and subjects' and mothers' medical records. Exclusion criteria included mother's excessive drug or alcohol abuse during pregnancy; grade III–IV intraventricular hemorrhage; periventricular leukomalacia; hypoxic-ischemic encephalopathy; lung disease or brochopulmonary dysplasia; body or heart malformations; chromosomal abnormalities; necrotizing enterocolitis that requires intestinal resection or complex feeding/nutritional disorders; defects or anomalies of forebrain, brainstem, or cerebellum; brain tissue dis- or hypoplasias; abnormal meninges; alterations in the pial or ventricular surface; or WM lesions. The parents of all neonate subjects gave written informed consents approved by the Institutional Review Board of UTSW.

### MRI data acquisition

#### MRI of *ex vivo* fetal brain specimens at around the middle trimester

Before each MRI scan, brain samples were kept immersed in a fixation solution for 48 h, after which the samples were transferred to PBS to wash out the fixative. The samples were kept immersed in PBS in a custom MR-compatible chamber. Three-dimensional multiple spin echo DTI was performed on *ex vivo* fetal brain samples using a 4.7T Bruker scanner with a 70 mm inner diameter Bruker volume coil. A multiple echo (number of echoes = 8) diffusion weighted images (DWI) sequence was adopted to improve the signal to noise ratio (SNR) (Huang et al., 2009). DWIs were acquired with b-value of 1,000 s/mm^2^ along seven linearly independent diffusion encoding directions with the following parameters: effective TE = 66 ms, TR = 0.8 s, FOV = 48–52/48–52/48–52 mm, imaging matrix = 128 × 72 × 72 (zero filled to data matrix = 128 × 128 × 128). The resultant image resolution was isotropic 280–350 μm. Two repetitions were performed to improve SNR. The total imaging time was ~20 h per brain.

#### MRI of *in vivo* neonate subjects

Each MRI of preterm and term neonates was acquired with a 3T Philips Achieva MR system (Cleveland) at Children's Medical Center at Dallas with an 8-channel SENSE head coil. The neonates were fed before the MRI scan and wrapped with a vacuum immobilizer to minimize motion. Extra foam padding was applied to reduce the sound of the scanner in addition to the earplugs and the earphones. The DTI imaging parameters were as follows: b-value = 1,000 s/mm^2^, 30 linearly independent diffusion encoding directions (Jones et al., 1999), one non-diffusion weighted (b0) image, TE = 78 ms, TR = 6,850 ms, in-plane FOV = 168 × 168 mm^2^, in-plane imaging resolution = 1.5 × 1.5 mm^2^, slice thickness = 1.6 mm, and number of slices = 60. The axial DWI image dimension was 256 × 256 after reconstruction. Two repetitions were performed to improve SNR. The total acquisition time was 11 min. DWI volumes that were corrupted due to artifacts or motion were replaced with another DTI repetition during postprocessing.

### DTI analysis and processing of the traced fibers from DTI tractography

#### DTI analysis

DWI data acquired from all subjects was processed offline using DTIStudio (Jiang et al., 2006) and TrackVis (Wang et al., 2007). DWIs for each subject were first corrected for eddy current distortions and motion artifacts by registering all the DWIs to the b0 image using a 12-parameter (affine) linear image registration with automated image registration (AIR) algorithm (Woods et al., 1998). Six independent elements of the 3 × 3 diffusion tensor were determined in every voxel by multivariate least-square fitting of DWIs (Basser et al., 1994). The reconstructed tensor matrix was diagonalized to obtain three eigenvalues (λ_1−3_) and eigenvectors (υ_1−3_). The corresponding FA value of each voxel was calculated based on eigenvalues (Pierpaoli and Basser, 1996) and the primary eigenvector (υ_1_) was used to infer the fiber orientation within the voxel. Whole brain deterministic fiber tracing was performed with TrackVis using the method of fiber assignment by continuous tracking (FACT) (Mori et al., 1999) with FA threshold = 0 (Takahashi et al., 2012) and turning angle threshold = 30°.

#### The traced brain fibers filtered at different lengths for 20, 35, and 40 PMW brains

To further demonstrate the dynamic profile of developing fetal brains, for a certain fetal brain at 20, 35, or 40 PMW, all brain fibers were filtered at different lengths, specifically 0, 1/8, 1/4, 3/8, and 1/2 of the anterior-posterior length L of a given fetal brain. That is, the fibers shorter than certain lengths were removed gradually to reveal the distribution of fibers with different relative lengths.

#### Twenty postmenstrual weeks fetal brain fibers categorized into two groups based on their terminal locations

All traced fetal brain fibers at 20 PMW were categorized into two groups based on their terminal locations in the cerebral wall. The cerebral wall of the human fetal brain is a laminated structure (e.g., Kostović et al., 2002; Vasung et al., 2010) and can be delineated into three distinctive layers, namely, cortical plate with marginal zone, subplate and the inner layer, based on DTI contrasts (e.g., Huang et al., 2009, 2013; Huang, 2010). Group1 and group2 fibers can be categorized based on the fiber terminal locations in the subdivided cerebral wall layers. Fibers with both terminals located in the cortical plate were defined as group1 fibers. For group2 fibers, one of the terminals is located in the inner layer of the fetal brain cerebral wall, and the other terminal is located either in the cortical plate or inner layer.

### Network construction

**Figure 1:**
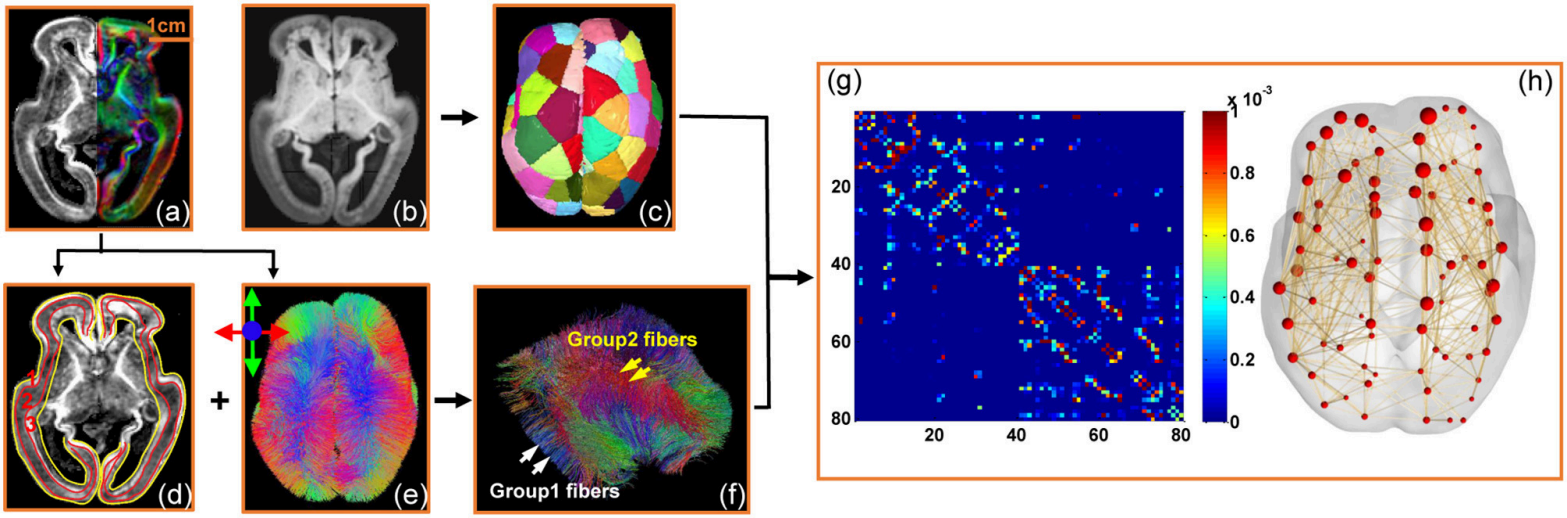
Flow chart demonstrating the data analysis process to quantify structural connectome of the human fetal brain with DTI data from a typical 20 PMW fetal brain. **(a)** The high resolution FA map on the left and DTI direction-encode colormap on the right; **(b)** the averaged DWI image; **(c)** parcellation of the fetal brain into evenly distributed regions; **(d)** segmentation of the cerebral wall into three layers based on the contrasts of FA map; **(e)** superior view of whole brain fibers based on DTI tractography with red, blue and green indicting left-right, superior-inferior, and anterior-posterior, respectively; **(f)** categorization of group1 and group2 fibers based on the fiber terminal locations in the cerebral wall (details in Figure 5 below); **(g)** the connectivity matrix of the structural network of this 20 PMW fetal brain with parcellated brain regions in **(c)** as nodes and traced brain fibers in **(e)** or **(f)** as edges; and **(h)** the three-dimensionally reconstructed structural network based on the connectivity matrix **(g)**. Scale bar is displayed in **(a)**.

The flow chart demonstrating the structural network construction of a fetal brain is shown in Figure 1.

#### Network node definition

**Figure 2:**
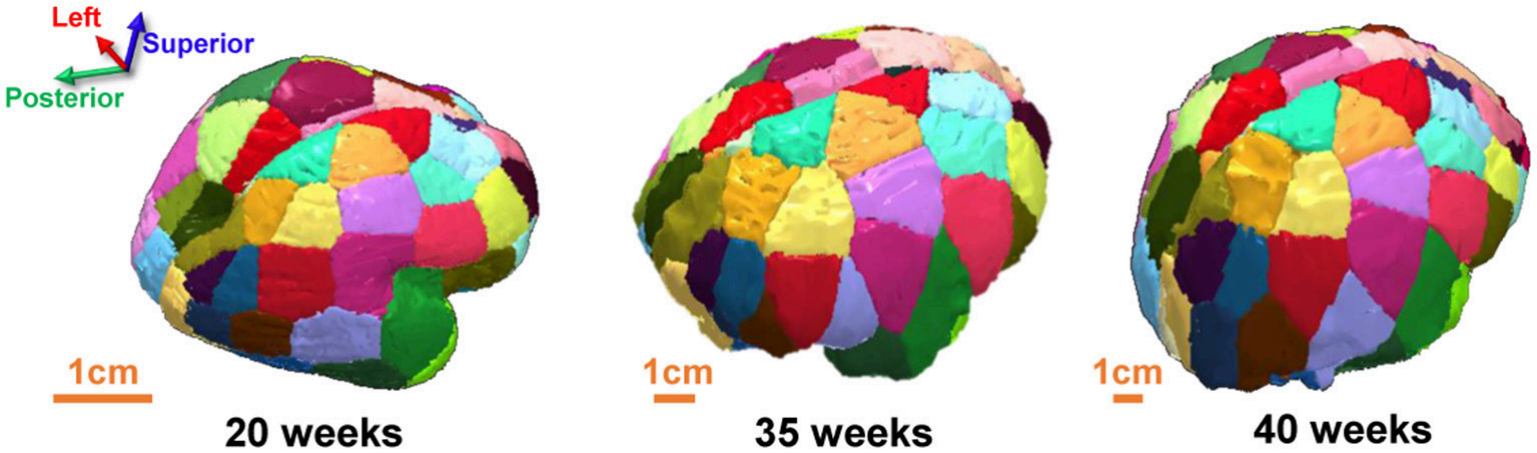
Posterodorsal view showing consistent brain parcellation of a typical 20 PMW **(Left)**, 35 PMW **(Middle)**, and 40 PMW **(Right)** fetal brain.

The cerebral wall of each *ex vivo* brain and the cerebral cortex of each *in vivo* subject was segmented using the following procedure. The subcortical structures were removed manually to obtain the cerebral wall of each *ex vivo* brain using ROIEditor (http://www.MriStudio.org). The cerebral cortex of each *in vivo* subject was obtained by segmenting the b0 brain using SPM8 (http://www.fil.ion.ucl.ac.uk/spm/software/spm8/). The cortical surface of 20 PMW brain is smooth and lacks anatomical landmarks. To our knowledge, a digital fetal brain atlas that can be used to parcellate the 20 PMW fetal brains does not yet exist. In addition, for network metric comparisons across brains at different postmenstrual ages, a consistent cortical network parcellation is needed. Due to those factors, a template-free parcellation scheme (Leopardi, 2006) previously used for baby brain parcellation (Tymofiyeva et al., 2012) was applied to evenly parcellate each hemisphere into 40 different regions with similar size. To ensure uniformity between hemispheres, left and right hemispheres were manually extracted for each subject from their respective b0 map and the template-free parcellation was run on each hemisphere separately for each subject. A recursive zonal equal area sphere partitioning was used to divide the cerebral wall (for 20 PMW brains) or cerebral cortex (for 35 or 40 PMW brains) into nodes of equal area. Briefly, a unit sphere was first divided into regions of equal area and the set of center points of the regions was determined that served as the node reference point. The sphere was then scaled to each hemisphere and every voxel on each hemisphere was assigned to the closest node reference point. Such an approach resulted in nodes of similar sizes that were consistent between hemispheres within a subject and across subjects of three age groups, and was obtained without any anatomical constraint. Of note, the cortical regions of brains t 35 and 40 PMW were dilated by five voxels in order to allow traced WM fibers to reach the cortical nodes (Jeon et al., 2015). As can be observed in Figure 2, the segmented nodes were consistent between the hemispheres for each brain and consistent across the brains of each subject at different postmenstrual ages.

#### Network edge definition

Two nodes were considered to be structurally connected if there existed at least one traced fiber with two end-points located in these two nodes. There were relatively more traced fibers generated for the *ex vivo* brain than the *in vivo* brain as the *ex vivo* DTI datasets had higher resolution than the *in vivo* ones. In addition, there were relatively more fibers connecting nodes with larger number of voxels. To take these effects into account, the weight (*w*_*i, j*_) of edge connecting node i and node j was calculated as the number of streamlines connecting the two nodes scaled by the voxel size and number of voxels of node i and node j. FA was also used to scale the edge weight, so that the edge weight was computed as:

(1) $$\begin{array}{l} Edge\;weight\;w_{i,j}=\\ \;\frac{\left(Number\;of\;fibers\;connecting\;node\;i\;and\;node\;j\right)*Voxel\_size}{Number\;of\;voxels\;in\;node\;i+Number\;of\;voxels\;in\;node\;j}*FA \end{array}$$

To minimize spurious connections due to noise, two nodes were connected only if there were at least 10 traced fibers between the two nodes and the length of each fiber was greater than 4 mm for the *ex vivo* brains, and greater than 10 mm for the *in vivo* subjects. As a result, we constructed a weighted network matrix for each participant, which was represented by a symmetric 80 × 80 matrix. In addition to comparing dynamics of structural connectivity with development, the fibers of the *ex vivo* 20 PMW brains were categorized into group1 and group2 fibers (see previous section). This procedure resulted in an additional two symmetric 80 × 80 matrices for each *ex vivo* 20 PMW brain, for computation of network measures contributed by group1 fibers and group2 fibers.

### Network analysis

Various graph metrics were estimated to characterize the topological organization of structural network of fetal brains at 20, 35, and 40 PMW. The graph metrics were calculated at various sparsity thresholds, and included the network strength, network shortest path length, global and local efficiency, normalized shortest path length (normalized Lp), normalized clustering coefficient (normalized Cp), and small-worldness (normalized Cp divided by normalized Lp). All network analysis was performed using GRETNA (Wang et al., 2015) with details below.

The strength of node was defined as the sum of the edge weights (*w*_*i, j*_) connected to the node. Network strength of each weighted network graph (*G*) was computed as average of the strengths across all the nodes (*N*) computed as:

(2) $$Strength\;(G)=\frac{1}{N}\sum_{i\in \;G}S(i)$$

where *S*(*i*) is the sum of the edge weights (*w*_*i, j*_) linking to node *i*.

Network integration of a graph *G* quantifying the network's ability to transfer information in parallel was computed as network shortest path length (*Lp*) as:

(3) $$Lp\;\left(G\right)=\frac{1}{N(N-1)}\sum_{i\neq j\in \;G}L_{i,j}$$

where *L*_*i, j*_ is defined as the length of the path for nodes *i* and *j* with the shortest length, and length of each edge was defined as the inverse of the edge weight (*w*_*i, j*_).

The global efficiency (*E*_*glob*_ (G)) (Latora and Marchiori, 2001) of the graph *G* measures the efficiency of the parallel information transfer in the network and was computed as the reciprocal of the shortest path length represented as:

(4) $$E_{glob}\;\left(G\right)=\frac{1}{N(N-1)}\sum_{i\neq j\in \;G}\frac{1}{L_{i,j}}$$

The local efficiency (*E*_*loc*_(G)) of the graph *G* provides information about the fault tolerant ability and reveals the efficiency of the communication among first neighbors of the node *i* when it is removed. *E*_*loc*_ (G) is computed as:

(5) $$E_{loc}\;\left(G\right)=\frac{1}{N}\sum_{i\in \;G}E_{glob}G\left(i\right)$$

where *G*_*i*_ is the subgraph of the nearest neighbors of node *i*.

A network *G* is small-world if the network shortest path length is similar to degree-matched random network but has a higher clustering coefficient than the degree-matched random network (Watts and Strogatz, 1998). In this study, 100 degree-matched random networks were computed for each graph *(G)* to minimize the variance in the degree-matched random network (Prettejohn et al., 2011). Normalized clustering coefficient (γ) and normalized shortest path length (λ) was computed by normalizing each graph's $C_{p}^{real}$ (*G*) and $L_{p}^{real}$ (*G*) by $C_{p}^{random}$ (*G*) and $L_{p}^{random}$ *(G)* respectively. Small-worldness (σ) of the graph *G* is defined as a ratio between normalized clustering coefficient and normalized shortest path length, noted as σ = γ/λ. For a network to be small-world, γ > 1 and λ ≈ 1.

### Statistical analysis

Various network sparsity thresholds, between 0.01 and 0.1 and increasing in steps of 0.01 (10 thresholds) (e.g., Achard and Bullmore, 2007; Bassett et al., 2008), were used to evaluate the contributions of group1 and group2 connections to the structural networks of 20 PMW fetal brains and to evaluate relationship of fetal brain network measures and the postmenstrual ages. To justify the threshold range of 0.01–0.1, we expanded threshold range to 0.01–0.4. As shown in Supplementary Figures 1, 2, most of the network properties are nearly stable across the extended threshold range of 0.1–0.4 (Supplementary Figure 1A). Moreover, the age-related network property changes presented in the Results section below are consistent to those with expanded threshold range 0.01–0.4 (Supplementary Figure 2). Therefore, the threshold range of 0.01–0.1 was adopted for group comparison of network measures and a representative sparsity threshold of 0.05 (median sparsity threshold value for the range of 0.01–0.1) was used for the age-related network measures presented below.

#### Evaluation of contributions of group1 fibers only, group2 fibers only and combined group1 and group2 fibers to the structural networks of 20 PMW:

To evaluate the contribution of different types of fibers to the structural brain network measures, comparisons were performed among three groups of fibers (group1 fibers, group2 fibers, and combined fibers) using non-parametric ANOVA with *post-hoc* non-parametric 2-sample *t*-tests when needed (*p* < 0.05 after correcting for multiple comparisons). For non-parametric 2 sample *t*-tests under different network sparsity thresholds, *p* < 0.05/(number of sparsity thresholds) was considered significant after Bonferroni correction (Bonferroni, 1936). No correction was applied to statistical comparisons of integrated measures.

#### Evaluation of relationship of fetal brain network measures and the postmenstrual ages

All neural fibers in 20 PMW fetal brain, namely, the combined group1 and group2 fibers, were used for statistical analyses of age-related network measures below. To test (i) the network measure significantly changes during 20–35 or 35–40 PMW, and (ii) the change rates of network measure are different between the periods of 20–35 and 35–40 PMW, a general linear model (GLM) as shown in the following equation was used.

(6) $$\begin{array}{l} \text{network }{\text{measures}}_{i,j,k}=\;\beta_{0,\text{i},\text{j},\text{k}}\;+\;\beta_{1,\text{i},\text{j},\text{k}}P_{\text{i}}\;+\;\beta_{2,\text{i},\text{j},\text{k}}\tau_{j}\\ \;+\;\beta_{3,\text{i},\text{j},\text{k}}\;({\text{P}}_{\text{i}}\;\tau_{\text{j}})\;+\;\varepsilon_{\text{i},\text{j},\text{k}} \end{array}$$

where network measures_*i,j,k*_ was defined as the values of network properties *P*_i_, at a specific time point τ_*j*_, and of subject *k*; β_0,i,j,k_ was the constant; β_1,i,j,k_, β_2,i,j,k_ and β_3,i,j,k_ represent the parameters to be estimated for *P*_*i*_, τ_*j*_ and *P*_*i*_ τ_*j*_, respectively; ε_i,j,k_ was the error term, satisfying identical independent distribution (i.i.d.) with standard deviation; *P*_i_ was one of the network properties, namely, network strength, global efficiency, local efficiency, shortest path length, small-worldness, normalized Lp, or normalized Cp; *i* was from 1 to 7, indicating *i*th network measures; τ_*j*_ was one of the time points, namely, 20, 35, and 40 PMW; *j* was from 1 to 3, indicating *j*th time points; *k* was from 1 to 10 at 20 PMW, 1 to 12 at 35 PMW, and 1 to 12 at 40 PMW. Statistical procedures were performed using R statistical software version 3.0.2 (http://www.r-project.org/).

#### Test of significant network measure changes during 20–35 or 35–40 PMW

The null hypothesis is that the network measure change rate is equal to 0 during the periods of 20–35 or 35–40 PMW. Rejection of the null hypothesis indicates significant network measure change at a specific time period. The GLM was reduced to include only the age variable τ_*j*_ with *j* = 1, 2 for 20–35 PMW and *j* = 2, 3 for 35–40 PMW as well as the error and constant term. Bonferroni correction (Bonferroni, 1936) for multiple comparisons was applied.

#### Test of the network measure change rates between the periods of 20–35 and 35–40 PMW

The null hypothesis is defined as the network measure change rate from 20 to 35PMW is equal to the change rate from 35 to 40 PMW for a specific network property. Rejection of the null hypothesis indicates a significant acceleration or deceleration of the network measure change rates from 20–35 to 35–40 PMW. The GLM was reduced to include only the age variable τ_*j*_ as well as the error and constant term. When β_2_ in the two time periods are equal, the null hypothesis will be rejected, indicating a significant alteration of the network measure change rates between the two time periods and temporally non-uniform network measure changes from 35–40 PMW compared to the changes during 20–35 PMW. Bonferroni correction for multiple comparisons was applied.

## Results

### Changing profile of fetal brain fibers during development

**Figure 3:**
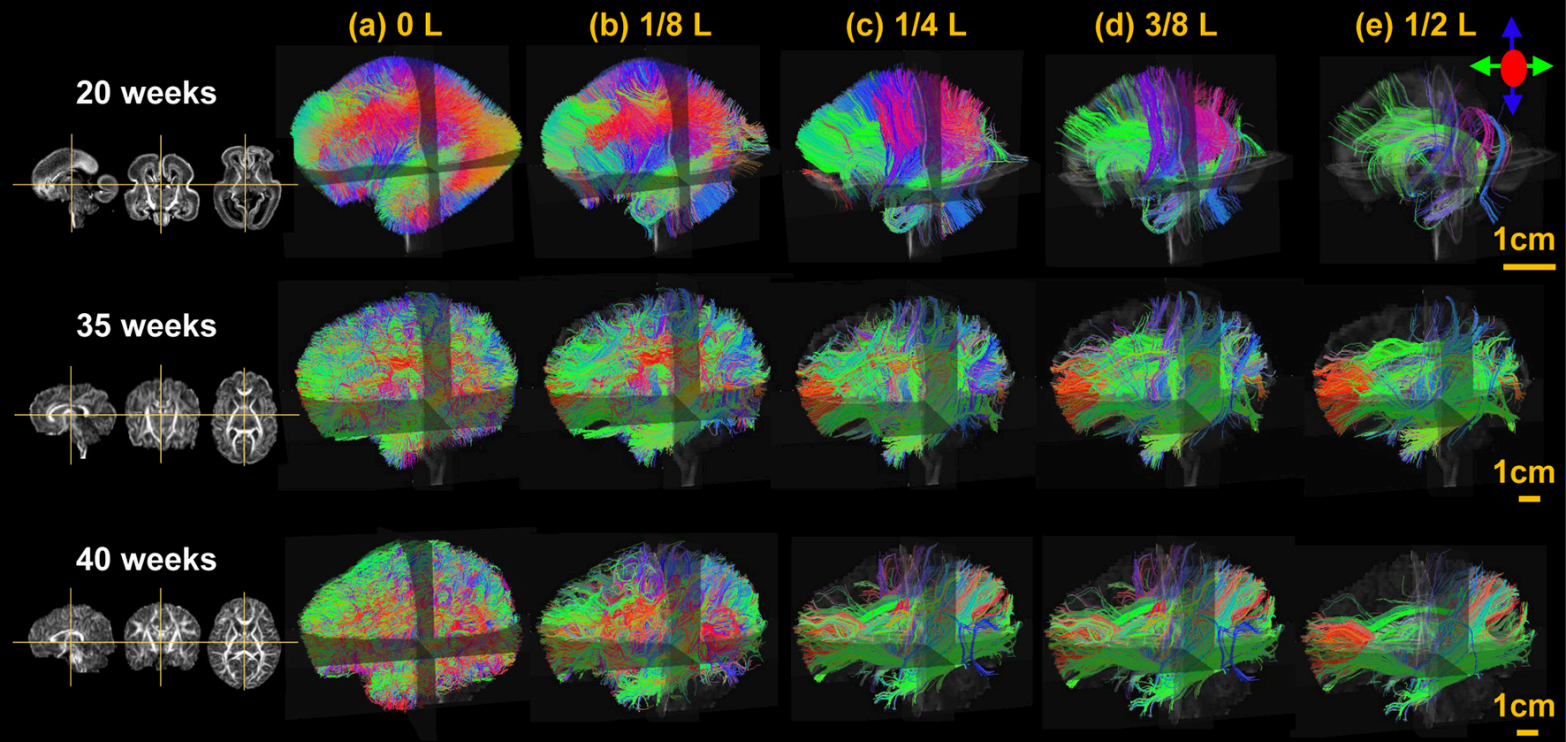
The lateral view of the three-dimensionally reconstructed whole brain fibers filtered at different lengths, namely 0 L **(a)**, 1/8 L **(b)**, 1/4 L **(c)**, 3/8 L **(d)**, and 1/2 L **(e)** of a typical 20, 35, and 40 PMW brain as shown in the upper, middle, and lower panels, respectively. L is the anterior-posterior length of each brain. On the left, the sagittal, coronal, and axial FA maps of each brain is displayed as anatomical guidance from left to right.

From Figure 3, it is clear that the fetal brain fibers become denser from 20 to 40 PMW, suggesting more brain fibers appearing in this developmental period. The fetal brain fiber profile characterized by the distribution of the fibers with different relative lengths is also distinctive across these postmenstrual ages. Specifically, at 20 PMW, the fetal brain is rich of fibers but most of them are short ones with almost no long fibers connecting to occipital lobe. During fetal development from 20 to 40 PMW, it is apparent from Figure 3 that more long-range fibers connecting between occipital lobe and distal frontal or temporal lobes appear at 35 and 40 PMW.

### Twenty postmenstrual weeks fetal brain cerebral wall and categorization of the traced brain fibers based on their terminal locations in the cerebral wall

#### Fetal brain cerebral wall

**Figure 4:**
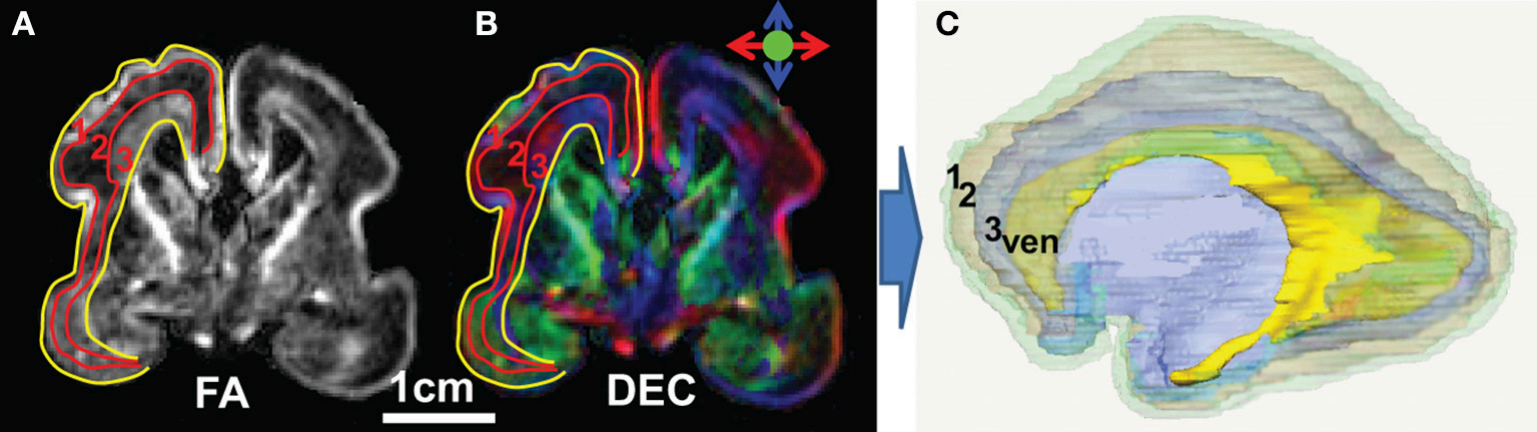
Subdivision of the 20 PMW human fetal brain cerebral wall into three layers. **(A)** Coronal image of the FA map; **(B)** coronal image of the direction-encoded colormap map; and **(C)** three-dimensionally reconstructed three layers of a 20 PMW fetal brain. In **(A)** and **(B)**, red curves separate the three layers in the cerebral wall based on FA contrast and yellow curves separate the cerebral wall from others. In **(C)**, transparent layer 1 (green), layer 2 (light brown), and layer 3 (blue) are cortical plate (with marginal zone), subplate and inner layer (Huang et al., 2013), respectively. Ventricle (yellow) is also displayed in **(C)** as anatomical reference. ven: ventricle.

As can be observed from the coronal image of FA map and direction-encoded colormap of a representative 20 PWM fetal brain on the left and middle panels of Figures 4A,B, the fetal brain cerebral wall can be subdivided into three distinctive layers based on the contrasts of FA map, namely, cortical plate with marginal zone (layer 1), subplate (layer 2), and an inner layer (layer 3). The cortical plate is the future cerebral cortex. The segmented three layers of the 20 PMW fetal brain were reconstructed three-dimensionally, shown in the right panel of Figure 4C.

#### Categorization of the traced brain fibers based on their terminal locations in the cerebral wall

**Figure 5:**
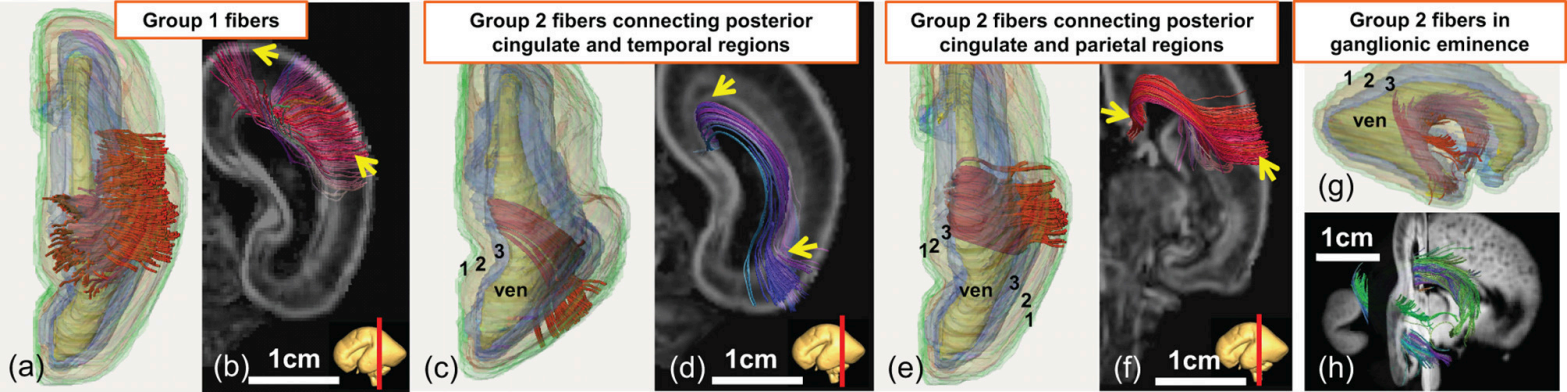
Demonstration of three-dimensionally reconstructed group1 or group2 neural fibers categorized based on the terminal locations in a 20 PMW fetal brain. Superior view of three-dimensionally reconstructed group1 fibers (red) with subdivided three layers is shown in **(a)**. The corresponding group1 fibers displayed on top of a coronal slice of the FA map is shown in **(b)**. Superior view **(c, e)** and lateral view **(g)** of different types of group2 fibers (red) with reconstructed segmented three layers as anatomical reference are shown in **(c,e,g)**, respectively. The corresponding group2 fibers displayed on top of the coronal slices **(d,f)** of FA map and sagittal and coronal slice **(h)** of the aDWI image are also shown. See Figure 4 legend for the color scheme of three cerebral wall layers and abbreviation.

As demonstrated in Figure 5, the terminal locations of the 20 PMW fetal brain fibers are distinctive and the traced fibers can be categorized into two groups based on their terminal locations. Both terminals of group1 fibers are located in the cortical plate (layer 1), as shown in Figures 5a,b. The majority of brain fibers are group2 fibers with one of the fiber terminals located in the inner layer. Three types of group2 fibers are demonstrated in Figures 5c–h. For these group2 fibers, one of the terminals is located in the inner layers and the other terminal is located in the cortical plate (layer 1) (Figures 5c–f) or the inner layer (layer 3) (Figures 5g,h).

### Network measures contributed by different groups of fibers and small-world property in the 20 PMW fetal brain structure connectome

#### Network measures contributed by different groups of fibers

**Figure 6:**
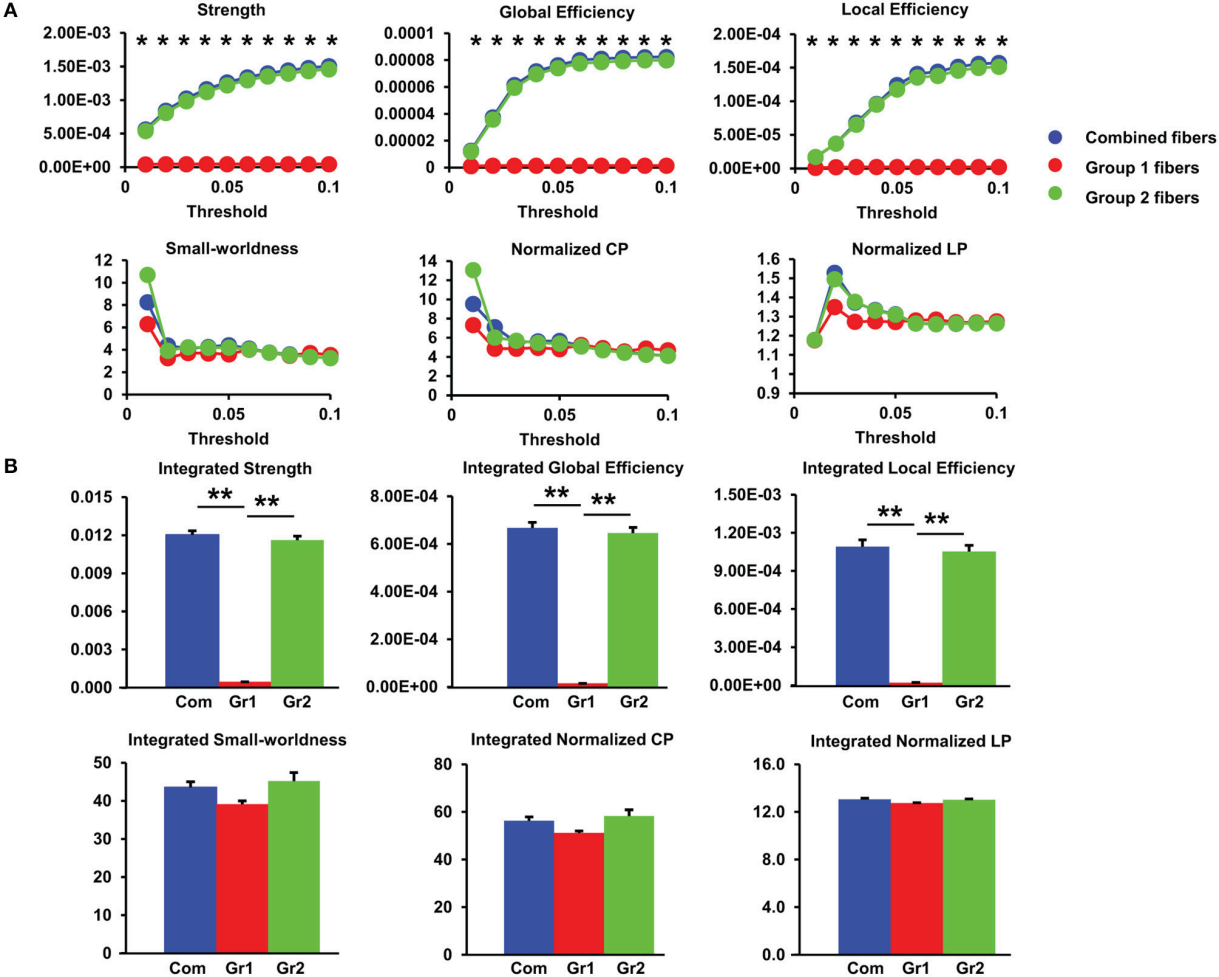
**(A)** Group differences in network measures among group1, group2, and combined fibers under different sparsity thresholds for 20 PMW fetal brain structural connectome. Asterisks: significant group differences with non-parametic ANOVA at *p* < 0.05/10 (Bonferroni-corrected). **(B)** Differences in integrated global network measures contributed by three groups of fibers. ^**^p < 0.01. The error bars indicate standard deviation. Com: combined group1 and group2 fibers; Gr1: group1 fibers; Gr2: group2 fibers.

The network measures contributed by group1 fibers only, group2 fibers only, and combined group1 and group2 fibers at different thresholds are shown in Figure 6A. The integrated values of these network measures are shown in Figure 6B. Figure 6 demonstrates that the network strength and efficiencies are mainly contributed by group2 fibers while small-worldness properties are more evenly contributed by both groups of fibers. From Figure 6A, the contribution of group2 fibers to network strength and efficiencies are significantly larger than that of group1 fibers at all sparsity thresholds from 0.01 to 0.1 (Bonferroni-corrected *p* < 0.05). From Figure 6B, the integrated network strength, global efficiency, and local efficiency contributed by the group1 fibers are much less than those by the group2 fibers. It is clear that the group1 fibers have very limited contribution to these network properties in 20 PMW fetal brains. Most of these network properties are contributed almost exclusively by the group2 fibers.

#### Small-world property of 20 PMW fetal brain structural connectome

Small-word property is prominent at 20 PMW fetal brain structural connectome. As shown in Figure 6A, structural networks with group1 fibers, group2 fibers or combined fibers at all sparsity thresholds show characteristic small-worldness (normalized Cp/normalized Lp > 1) with the normalized Cp greater than 1 and the normalized Lp close to 1, indicating the appearance of small-world organization as early as 20 PMW.

### Fetal structural network development from 20 to 40 PMW

#### Network metric changes from 20 to 40 PMW

**Figure 7:**
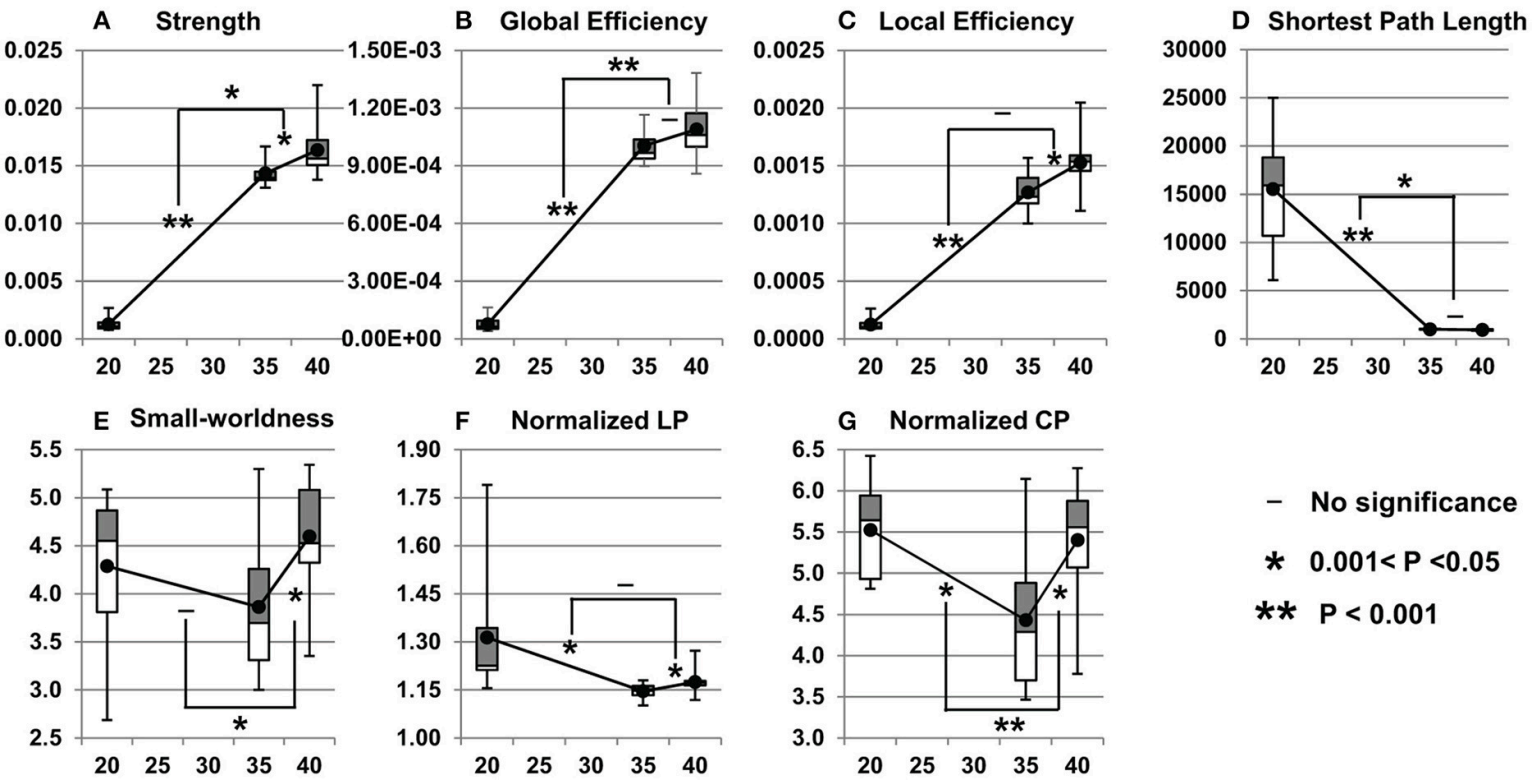
Cross-sectional network property (**A**: strength; **B**: global efficiency; **C**: local efficiency; **D**: shortest path length; **E**: small-worldness; **F**: normalized LP; **G**: normalized CP) changes during 20–35 and 35–40 PMW. Network measures from all subjects at a certain postmenstrual age are shown as boxplots. The averaged network measures of all the subjects at each time point are shown as solid black dots. The symbols on the bridges connecting network measure changing lines during 20–35 and 35–40 PMW demonstrate the statistical significance of the changing rate differences. That is, the significance of acceleration or deceleration of network measure changes during this two developmental periods. All *p*-values were calculated after Bonferroni correction. All network measures were obtained at the sparsity threshold of 0.05.

As shown in Figure 7, the fetal brain structural network gets stronger and more efficient from 20 to 40 PMW. More than 10-fold increases were found in network strength, global efficiency, and local efficiency (Figures 7A–C), reflecting dramatic structural architecture reconfiguration from the middle fetal stage to birth. The significant increases of network strength in both 20–35 and 35–40 PMW shows that the structural network continuously gets stronger in both periods. The significant increases of local efficiency in both 20–35 and 35–40 PMW indicate a continuous enhancement in efficiency of the local information transfer between neighboring nodes in both periods. As shown in Figure 6, the small-world organization already appears at 20 PMW fetal brain. Figure 7E shows that the small-world organization is also found in the 35 and 40 PMW fetal brains.

#### Network metric change rates in 20–35 and 35–40 PMW

Non-uniform network property changes were found in the two fetal developmental periods of 20–35 and 35–40 PMW. With the statistical comparisons of the network measure change rates between these two developmental periods, Figures 7A,B demonstrate that the network strength and global efficiency increase more rapidly in 20–35 PMW than in 35–40 PMW. Shortest path length drops significantly and rapidly in the period of 20–35 PMW with no significant decrease of shortest path length in the period of 35–40 PMW (Figure 7D), suggesting that major integration in brain structural network organization occurs at the late 2nd trimester and early 3rd trimester, but not in the late 3rd trimester.

## Discussion

Significant age-dependent increases of network strength, global efficiency, and local efficiency were found from 20 to 40 PMW, in parallel to the growth of long-range fibers during fetal brain development. The contribution of two groups of neural fibers to structural brain connectome at the middle fetal stage has been revealed. In the middle fetal stage, the brain WM fibers serving as the brain connectivity substrate exclusively in postnatal structural connectome are still scarce. The measurements of contribution of two distinctive groups of brain fibers to fetal structural connectome at 20 PMW may shed light on possible mechanism of critical yet unknown structural architecture maturation in the middle fetal stage. Small-worldness property was found in fetal brains as early as 20 PMW. In addition, more rapid increases of network strength and global efficiency in 20–35 PMW than in 35–40 PMW suggests dramatic structural reconfiguration from the middle-fetal stage to the middle 3rd trimester. Since it is generally difficult to acquire high resolution DTI data for fetal brains at 20 and 35 PMW, to our knowledge, this study represents the first record of quantified fetal brain structural connectome development from as early as 20 PMW.

### The fetal brain structural connections grow stronger and more efficient, possibly underlaid by the growth of long-range fibers

#### Stronger and more efficient structural network from the middle fetal stage to birth

We found that fetal brain structural configuration becomes stronger and more efficient from 20 to 40 PMW. As shown in Figures 7A–C, the network strength, global efficiency, and local efficiency increase dramatically and significantly during fetal brain maturation in this period. Studies on functional connections (Fair et al., 2008; Supekar et al., 2009; Hagmann et al., 2010; Power et al., 2010) suggested that the emergence of the human brain functional connectome is related to important structural developmental events. The structural network changes observed in the present study are consistent to the findings (van den Heuvel et al., 2015; Cao et al., 2017) that the functional networks get stronger and more efficient during the preterm brain development. Specifically, based on resting-state functional MRI data of the preterm brains in the 3rd trimester, it was found that the integration capacity of the functional connectivity increased from 30–40 weeks and the functional connectome became more efficient (van den Heuvel et al., 2015). Significant functional connectivity strength increases from 31 to 42 weeks were also found in our previous study (Cao et al., 2017).

#### Strengthened structural connectome possibly underlaid by the increasing long-range fibers

In our study, the most apparent changes of structural connectivity among the brains at 20, 35, and 40 PMW are the age-dependent growth of long-range fibers. There are more long-range association fibers connecting occipital and frontal lobes in 35 and 40 PMW fetal brains than those in 20 PMW fetal brains. The prominent development of long-range connectivity has also been found in *in-utero* fetal brains during 21–37 week (Jakab et al., 2014) and 24–38 week (Thomason et al., 2015), besides increases of cross-hemispheric functional connectivity strength during 24–38 weeks (Thomason et al., 2013). Previous studies (Kostović and Jovanov-Milošević, 2006; Huang et al., 2009; Vasung et al., 2010; Takahashi et al., 2012; Huang and Vasung, 2014) suggested that long-range association WM fibers, including uncinate, inferior longitudinal, and inferior fronto-occipital fasciculus, become apparent at 20 PMW, but are still quite immature and have not extended to the cortical regions they connect. Therefore, short-range fibers at 20 PMW fetal brains remain dominant (Takahashi et al., 2012) and play a major role in the fetal brain structural configuration. The dramatic growth of the major long association WM fibers from 20 to 40 PMW, as observed in Figure 3, could underlie the dramatic increases of network strength and efficiencies shown in Figures 7A–C.

### Network strength and efficiency increase more rapidly during 20–35 PMW than during 35–40 PMW

It was found that the network strength and global efficiency increase more rapidly during 20–35 PMW than 35–40 PMW (Figure 7). More rapid network property increase in 20–35 PMW may be related to more dynamic neuronal activities in this period including interaction of neurons to form bran circuits. It has been suggested that the radial glial fibers gradually transform to corticocortical fibers and WM astrocytes around 30–31 weeks (Xu et al., 2014), also contributing to enhanced network properties in this period. Furthermore, as can be observed from Figure 3, the major long association fibers connecting occipital region to frontal or temporal region grow more dramatically during 20–35 PMW than 35–40 PMW. These more dramatically increased long-range brain fibers in 20–35 PMW can increase the fiber count between certain paired regions, and could improve brain wiring efficacy in this period (Hagmann et al., 2008, 2010; Greicius et al., 2009; Honey et al., 2009; van den Heuvel et al., 2009).

### Small-world property exists in 20 PMW fetal brains

Figures 6, 7 show that as early as the middle fetal stage (20 PMW), the structural brain network is already characterized with small-world property. During the second trimester, neurons migrating along the radial glial fibers arrive in the cortical plate to form brain circuits through the maturational processes including dendritic arborization, axonal growth, and cellular differentiation (Rakic, 1972, 1995; Sidman and Rakic, 1982; Kostović and Rakic, 1984, 1990). These immature brain circuits could contribute to the small-world property of the fetal structural network at 20 PMW. As elaborated in the next section below, the 20 PMW fetal brain is also characterized by connections contributed by fibers that are possibly not fetal WM. These are group2 fibers demonstrated in Figure 5. These group2 fibers may contribute to the small-world property of the fetal structural networks. The characteristic small-world organization has not been reported for the fetal brain as early as 20 PMW previously, but has been consistently found in the structural and functional connectome of preterm or term-born neonate brains from early 3rd trimester to term (Doria et al., 2010; Ball et al., 2014; Brown et al., 2014; van den Heuvel et al., 2015; Cao et al., 2017).

### The brain fibers contributing overwhelmingly to well-established network properties in 20 PMW fetal brains might be non-WM fibers

Figure 6 shows that network strength and efficiencies of 20 PMW fetal brains are mainly contributed by group2 fibers, suggesting group2 fibers may lay the foundation for early fetal brain circuit formation and network maturation. Group1 fibers, defined as the brain fibers with both terminals located in the cortical plate, are likely short-range corticocortical fetal WM tracts. The group2 fibers, connecting between two cerebral wall regions with one terminal located in the inner layer, may not be the fetal WM tracts. Radial glial scaffold (Rakic, 1972, 1995; Sidman and Rakic, 1973), as neuronal migration pathways from the location close to ventricle to the cortical plate, has been well-characterized by dMRI tractography (Huang et al., 2009; Vasung et al., 2010; Takahashi et al., 2012; Huang and Vasung, 2014; Xu et al., 2014). Figures 5c–f show that a few group2 brain fibers are possibly part of the glial scaffold with pathways from the location close to ventricle to the cortical plate, but are not precisely “radially” oriented. Such non-radial glial scaffold pattern has been found in cellular studies (e.g., Lui et al., 2011). As shown in Figures 5g,h, some group2 fibers are located in the ganglionic eminence, also observed in our previous publication (Huang et al., 2006). It should be noted that the cellular properties of both group1 and group2 brain fibers cannot be determined with these fibers obtained by DTI tractography and categorization based on their terminal locations. DTI cannot provide the direct evidence of neural components to distinguish which fibers are WM (myelinated or unmyelinated axons) and which fibers are not WM. The assumption that group1 fibers are WM fibers and group2 fibers are non-WM glial fibers needs to be further validated with investigations using other modalities such as immunohistochemistry. Nevertheless, the present study suggests the critical role of non-WM fibers in the very early emergence of fetal brain structural networks.

### Technical considerations, limitations, and future directions

Several issues need to be considered. Relatively high resolution DTI data of fetal brains at different cross-sectional ages, namely, 0.283^3^−0.353^3^ mm^3^ for 20 PMW fetal brains and 1.5 × 1.5 × 1.6 mm^3^ for 35 and 40 PMW brains, were obtained. The high resolution DTI could reveal more detailed structural connections of these fetal brains with DTI tractography. The much higher resolution for DTI of 20 PMW fetal brains is needed due to dramatic size differences of fetal brains from 20 to 40 PMW. For example, the average whole brain volume of the 20 PMW fetal brains in this study is 20.9 ml while the average whole brain volume of the 40 PMW brains is 395.8 ml. The resolution of postmortem fetal brain samples at 20 PMW has to be much higher than that of 35 or 40 PMW brains to delineate the comparable details of the neural structures inside the brain. Despite relatively large resolution differences, the number of voxels of the whole brain (around 120,000) for a representative 40 PMW brain is comparable to the number of whole brain voxels (around 119,000) of a representative 20 PMW fetal brain specimen with the imaging resolution used in the present study. The comparability of the whole brain voxel number across the developmental age made it possible for investigating network properties using consistent parcellation protocol and fiber tracing method across the ages. Secondly, the structural connections are “mathematically” traced streamlines based on DTI tractography and categorization of the group1 and group2 fibers are based on their terminal locations. Although it has been well-recognized that DTI tractography is capable of revealing major WM fiber anatomy of live adult human brains (e.g., Wakana et al., 2004; Catani and Thiebaut de Schotten, 2008), much less is known on dynamic human fetal brain WM despite that efforts have been made to validate the traced WM fibers with histology (e.g., Xu et al., 2014; Ouyang et al., 2015). FA threshold of 0 (Takahashi et al., 2012) was used for tracing all brain fibers to ensure that the traced fibers are not terminated due to small FA values. But it should be noted that the DTI primary eigenvector, which is the fiber tracing orientation, is more sensitive to noise at the regions with low FA values, leading to potential bias of traced fiber pathways. As discussed in the previous section, we assume that group1 and group2 fibers are WM and non-WM fibers, respectively. However, differentiation of WM from non-WM fibers (such as glial fibers) needs to be determined by investigations with other modalities such as immunohistochemistry. Thirdly, due to smooth cortical surface of 20 PMW fetal brains and lack of well-recognized digital fetal brain atlas, the fetal brains across three postmenstrual ages were parcellated using a template-free parcellation scheme that results in consistent nodes across the brains of different ages. Such parcellation approach has also been applied to other network studies on neonate brains (e.g., Tymofiyeva et al., 2012). Cross-sectional datasets were acquired with *ex vivo* brain specimens used for DTI of 20 PMW fetal brains and *in vivo* neonates recruited for DTI of 35 and 40 PMW brains. It has been found that fixation of postmortem brain does not alter FA, a relative diffusion metric measurement, but significantly change the absolute diffusion measures such as mean, axial or radial diffusivities (Sun et al., 2003, 2005). It is generally believed that fixation does not significantly alter DTI primary eigenvector, another important parameter that may affect structural connectome quantifications besides FA. Several MRI studies of postmortem fetal brain specimens (e.g., Huang et al., 2009; Takahashi et al., 2012; Xu et al., 2014; Yu et al., 2016) also suggested that organized structures such as connectional fibers in the postmortem fetal brain tissues could be well-delineated with diffusion MRI given good fixation of the brain tissues. Preterm birth has been associated with adverse neurodevelopmental outcomes (e.g., Woodward et al., 2006). Despite that, many studies of preterm infants (Fransson et al., 2007; Doria et al., 2010; Smyser et al., 2010; Cao et al., 2017) have been conducted to understand brain development before normal time of birth. Exposure to the extrauterine environment could be one of the factors affecting the network measures of 35 PMW brains. However, compared with the dramatic developmental factor, these effects would be relatively subtle (Bourgeois et al., 1989; Kostović, 1991). Future studies with larger sample size of both *in-utero* fetal brains and preterm neonates are needed to understand the network differences between *in utero* fetal brains and the preterm brains at the same PMW. Finally, it should be cautious to differentiate the results of cross-sectional network changes from those of longitudinal network development, as the results from cross-sectional studies include not only age-related changes but also individual differences.

These limitations and technical considerations above warrant the future investigations of fetal brain structural connectome development using longitudinal dMRI datasets from the same cohort of subjects and with a larger sample size. With the advances of imaging technologies, *in-utero* MRI holds the promises for revealing *in vivo* fetal brain networks at relatively early stages such as 20–28 PMW (see van den Heuvel and Thomason, 2016 for review).

## Conclusion

With high resolution dMRI of fetal brains at 20, 35, and 40 PMW, we quantified the structural network transitions and delineated the profile of whole brain neural fibers underlying the structural network architecture based on DTI tractography and graph theory analysis. We found that during 20–40 PMW human fetal brains developed into a much stronger and more efficient structural network and the network strength and efficiency increased faster in 20–35 PMW than in 35–40 PMW, possibly due to the growth of long-range association fibers. Small-world network property was found to exist as early as 20 PMW. At the middle fetal stage, non-WM neural fibers may be the major contributor of the structural network. Characterizing the normal fetal brain network development can enhance our understanding on the emergence and maturation of fetal brain structural connectome. The network properties of early developing brain may be used as potential imaging biomarkers for detecting altered network associated with neuropsychiatric disorders such as autism.

## Author contributions

HH designed the study; LS, MO, VM, SL, and HH performed research; LS, VM, MO, and QP analyzed data; LS, HH, VM, MO, and MS wrote the paper.

### Conflict of interest statement

The authors declare that the research was conducted in the absence of any commercial or financial relationships that could be construed as a potential conflict of interest.
