# Supplementary material for: Human Fetal Brain Connectome: Structural Network Development from Middle Fetal Stage to Birth
Source: Front Neurosci. 2017 Oct 13;11:561. doi: 10.3389/fnins.2017.00561 (PMC5645529; doi:10.3389/fnins.2017.00561)

Supplementary Material

Human fetal brain connectome: structural network development from middle fetal stage to birth

Limei Song[1, 3,†], Virendra Mishra[2,†], Minhui Ouyang[3], Qinmu Peng[3], Michelle Slinger[3], Shuwei Liu[1,*], Hao Huang[3,4,*]

[1]Research Center for Sectional and Imaging Anatomy, Shandong Provincial Key Laboratory of Mental Disorders，Shandong University School of Medicine, Jinan, Shandong, China

[2] Cleveland Clinic Lou Ruvo Center for Brain Health, Las Vegas, NV, United States.

[3]Radiology Research, Children's Hospital of Philadelphia, PA, United States

[4]Department of Radiology, Perelman School of Medicine, University of Pennsylvania, PA, United States.

† Limei Song and Virendra Mishra have contributed equally to this work.

Corresponding author:
Hao Huang, Ph.D.
3401 Civic Center Blvd,
Department of Radiology,
Children's Hospital of Philadelphia,
University of Pennsylvania,
Philadelphia, PA 19104
Tel: 267-425-1599
Email: huangh6@email.chop.edu

Shuwei Liu, Ph.D.
No.44 West Wenhua Road
Research Center for Sectional and Imaging Anatomy, Shandong Provincial Key Laboratory of Mental Disorders
Shandong University School of Medicine
Jinan Shandong 250012
Tel: 13853183162
Email: liusw@sdu.edu.cn

Supplementary Figure 1: (a) Group differences in global network measurements among group1, group2 and combined fibers under different sparsity thresholds from 0.01-0.4. Asterisks: significant group differences with nonparametic ANOVA at $p<0.05/40$ (Bonferroni-corrected). (b) Differences in integrated global network measurements contributed by three groups of fibers. **$p<0.01$. The error bars indicate standard deviation. Com: combined group1 and group2 fibers; Gr1: group1 fibers; Gr2: group2 fibers.

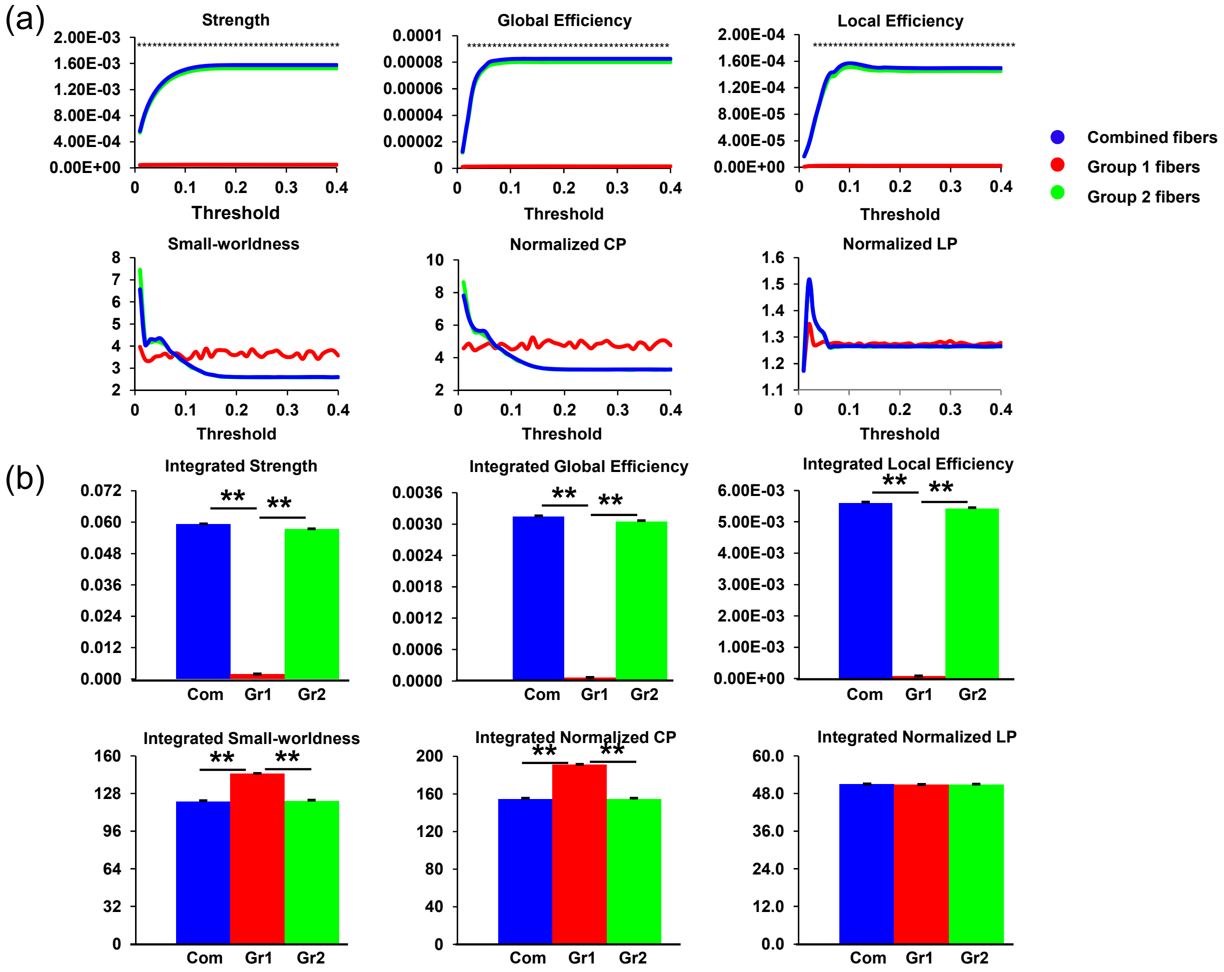

Supplementary Figure 2: Network property changes during 20-35PMW and 35-40PMW at the thresholds of 0.05 (a), 0.1 (b), 0.2 (c), 0.3 (d), and 0.4 (e). The averaged network metric measurements of all the subjects at each time point are shown as solid black dots. The symbols on the bridges connecting network measurement changing lines during 20-35 and 35-40PMW demonstrate the statistical significance of the changing rate differences. That is, the significance of acceleration or deceleration of network measurement changes during this two developmental periods. All p-values were calculated after Bonferroni correction.

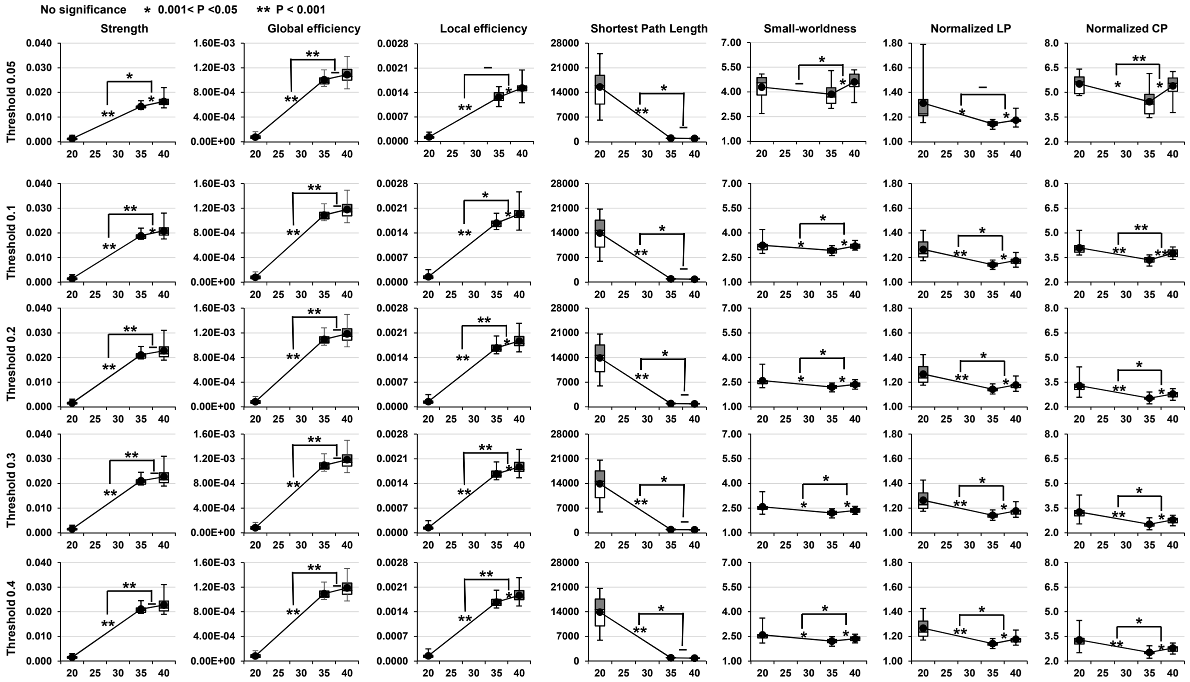

Supplement: Supplementary file 1 [file Presentation1.pdf]
